# Use of movement restrictions during an outbreak of COVID-19 in Selangor, Malaysia

**DOI:** 10.5365/wpsar.2020.11.3.008

**Published:** 2021-06-22

**Authors:** Anita Suleiman, Shaari Ngadiman, Mazliza Ramly, Ahmad Faudzi Yusoff, Mohamed Paid Yusof

**Affiliations:** a Ministry of Health Malaysia.; b Selangor State Health Department, Malaysia.; c Institute for Medical Research, Malaysia.; d Petaling District Health Office, Malaysia.

## Abstract

**Objective:**

Various public health and social measures have been used during the COVID-19 outbreak, including lockdowns, contact-tracing, isolation and quarantine. The objective of this manuscript is to describe outbreaks of COVID-19 in Selangor, Malaysia, the public health strategies used and the observed impact of the measures on the epidemic curve.

**Methods:**

Information on all confirmed COVID-19 cases in Selangor between 25 January and 28 April 2020 was obtained. Clusters were identified, and cases were disaggregated into linked, unlinked and imported cases. Epidemic curves were constructed, and the timing of movement control orders was compared with the numbers of cases reported.

**Results:**

During the study period, 1395 confirmed COVID-19 cases were reported to the Selangor Health Department, of which 15.8% were imported, 79.5% were linked and 4.7% were unlinked cases. For two main clusters, the number of cases decreased after control measures were instituted, by contact-tracing followed by isolation and home quarantine for the first cluster (*n* = 126), and with the addition of the movement control order for the second, much larger cluster (*n* = 559).

**Discussion:**

The findings suggest that appropriate, timely public health interventions and movement control measures have a synergistic effect on controlling COVID-19 outbreaks.

Severe acute respiratory syndrome coronavirus 2 (SARS-CoV-2) was first identified in Malaysia on 25 January 2020; three cases were notified, all of which were imported from Wuhan, China. On 30 January 2020, WHO declared coronavirus disease 2019 (COVID-19) a public health emergency of international concern. On 6 February, the first local transmission was reported in Malaysia in a close contact of a confirmed COVID-19 case who had returned from Singapore. The first case in Malaysia with neither a history of contact with a confirmed case nor travel to an affected area was reported on 12 March 2020. By 28 April 2020, Malaysia had reported 5851 confirmed cases and 100 fatalities.

Selangor is the most densely populated state in Malaysia, with a population of 5.8 million and a population density of 780.3 people/km^2^. It is situated in Peninsular Malaysia, bordering the capital, Kuala Lumpur, and the Federal Government Administrative Centre, Putrajaya. By mid-March 2020, there were more than 200 COVID-19 cases in Selangor, and the number increased to more than 1300 by mid-April 2020, largely due to two main clusters. The Malaysian Government instituted movement restrictions through a mandatory movement control order (MCO) under the Prevention and Control of Infectious Diseases Act 1988 and the Police Act 1967 to limit human movement from 18 March in an effort to prevent further COVID-19 cases.

A variety of containment strategies, used either in isolation or in combination, have been used for COVID-19, which can be broadly categorized as physical distancing measures, movement restrictions, public health measures and socioeconomic measures. (*1*) This paper describes the epidemiology and control measures used to control the outbreak of COVID-19 in Selangor, Malaysia, up to April 2020.

## Methods

This observational study included all COVID-19 cases reported in Selangor between 25 January and 28 April 2020. By that time, Selangor had reported 25% of all COVID-19 cases in Malaysia.

A confirmed case was defined as an individual with a positive test for SARS-CoV-2 by reverse transcriptase-polymerase chain reaction from nasopharyngeal swabs. We obtained demographic, clinical and exposure information from an online data collection form used by district health authorities in case investigation. Clusters were identified from detailed movement histories of confirmed cases and their contacts.

An epidemic curve was plotted, with the date of onset of illness used for symptomatic cases and the date of last exposure plus 5 days as the estimated “onset date” for asymptomatic cases. We defined cases as “imported” if they had travelled overseas in the 14 days before onset, as “linked” if the disease was acquired locally after a history of contact with a COVID-19 case and as “unlinked” for those with no history of contact with a confirmed COVID-19 case. Data were analysed in Microsoft Excel with SPSS version 26.

The control measures used during the period of measuring the epidemic curve are described.

### Ethics approval

The study protocol was reviewed and approved by the Medical Research and Ethics Committee, Ministry of Health Malaysia (NMRR-20–1043–54912 [IIR]).

## Results

As of 28 April 2020, 1395 confirmed COVID-19 cases had been reported to Selangor Health Department. Most (80%) were detected by contact-tracing, 13% were imported, 5% were detected by sampling of people with influenza-like illness or severe acute respiratory illness at sentinel surveillance sites, and 2% were found during routine passive case detection.

Most of the COVID-19 cases were in Malaysian citizens (85%) and males (59%). The age range was 1 month to 92 years (median, 35 years); 10.4% were aged < 19 years, 46.5% 19–39 years, 27.2% 40–59 years and 14.5% (*2*)60 years. Of the 1395 cases, 15.8% were imported, 79.5% were linked cases and 4.7% were unlinked cases.

**Figure 1:**
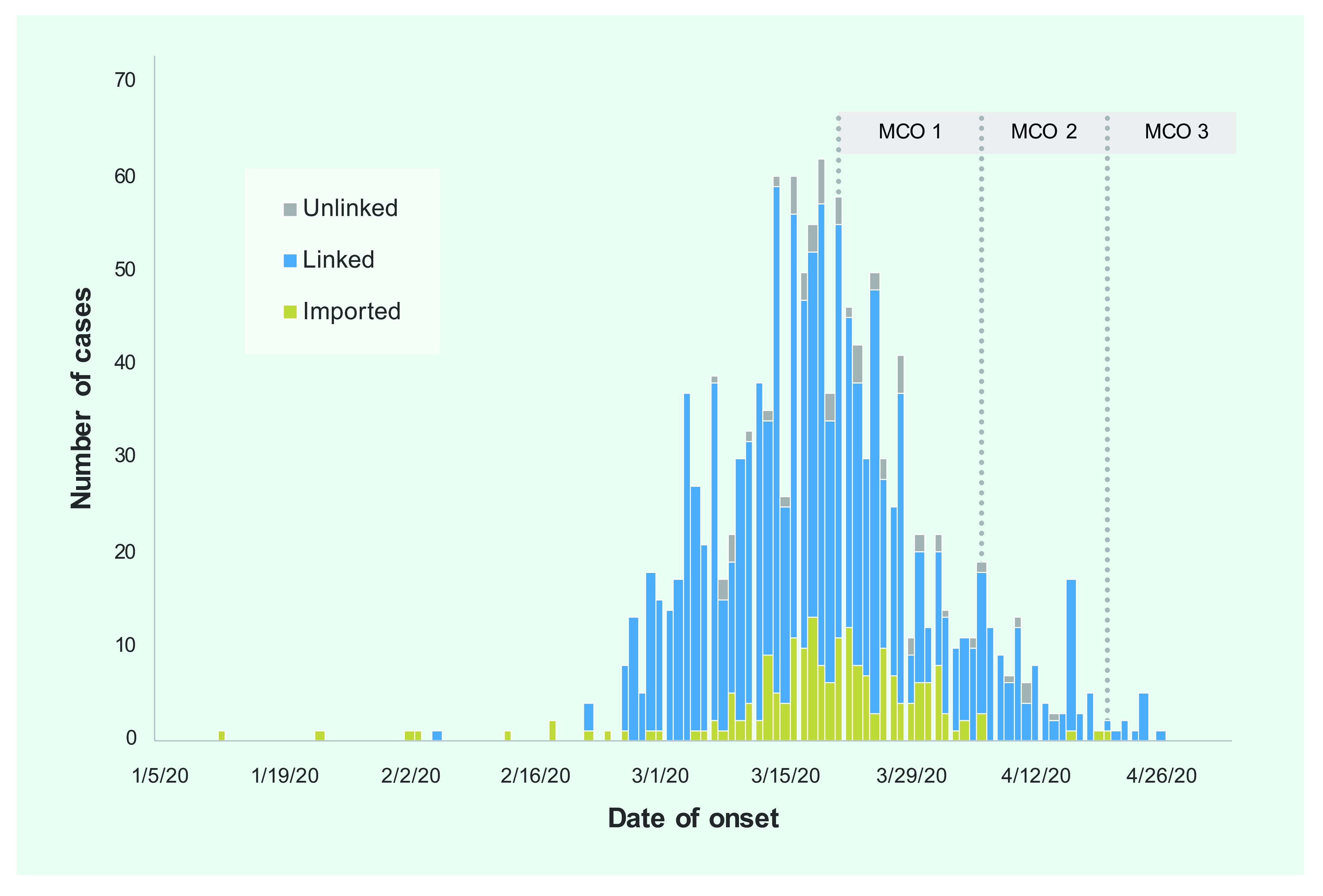
Epidemic curve of COVID-19 cases by importation and linkage between 5 January and 28 April 2020, Selangor, Malaysia (n = 1395)

The epidemic curve (**Fig. 1**) shows an exponential increase in the number of cases in Selangor from early March 2020, which peaked on 19 March, followed by a steady decline by 28 April.

[**insert** Figure 1]

Initial case detection and control measures included contact-tracing, isolation of cases and home quarantining of contacts of cases. Travellers and returning Malaysians with either symptoms or fever detected with thermal scanners at points of entry were tested for SARS-CoV-2. Those found to be positive were isolated in a designated COVID-19 hospital, while those found to be negative and/or asymptomatic were quarantined in designated hotels for 14 days from the date of arrival.

The increase in the number of linked cases after 22 February was due to a workplace cluster. Extensive case investigations revealed 126 confirmed cases among 1715 contacts, for an attack rate of 7.3%. This attack rate was higher among work-related contacts (18.7%, 56 of 300) than among family and social contacts (4.9%). The case with the earliest onset of illness, on 18 February, was identified as the primary case for this cluster and was imported from a neighbouring country. The largest potential exposure event was on 27 February, at a meeting with approximately 300 people. The number of cases in this cluster peaked on 29 February and then declined, in line with public health measures initiated on 29 February (**Fig. 2A**).

**Figure 2A:**
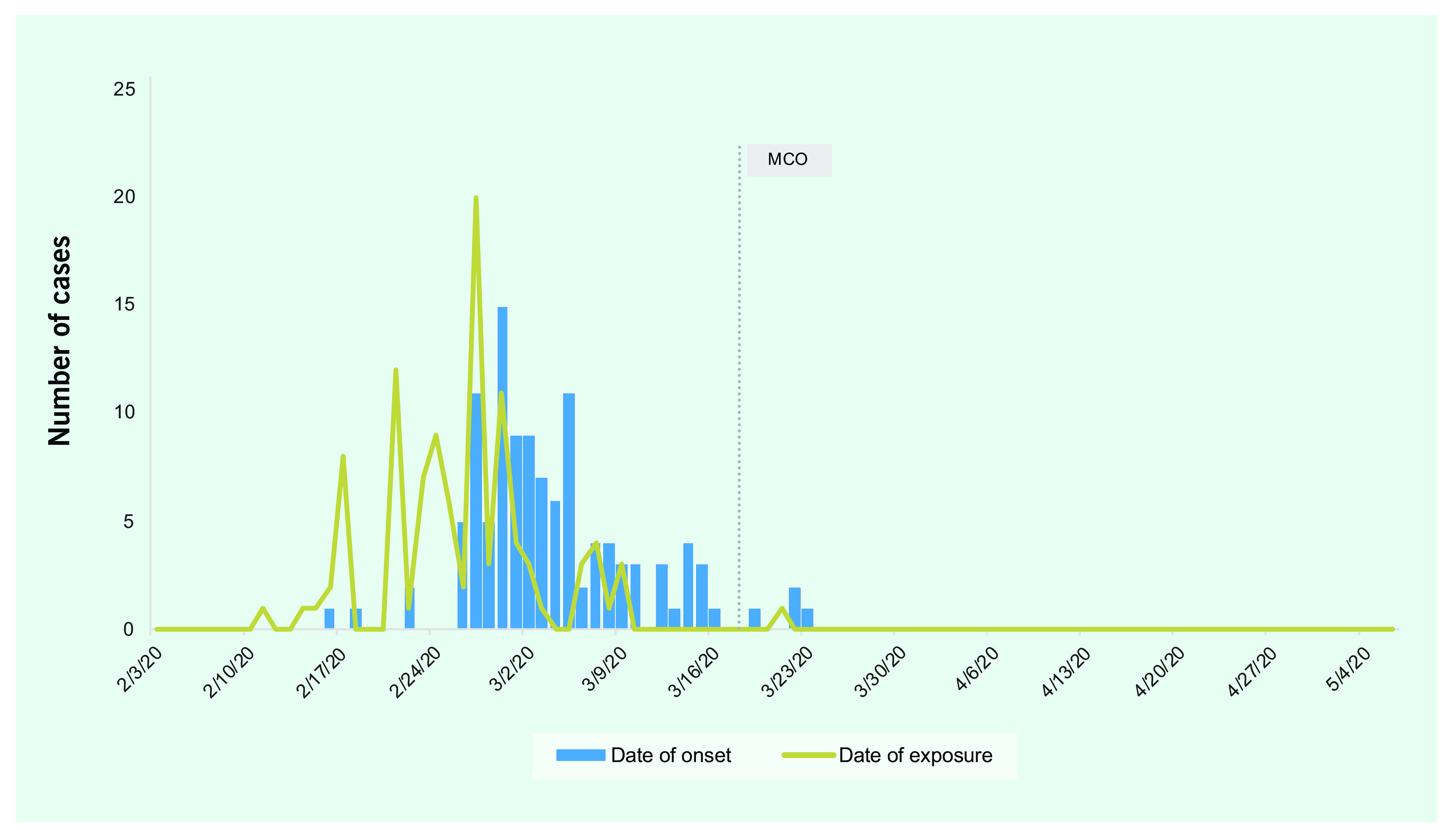
Distribution of cases by date of illness onset and date of exposure in a workplace cluster, Selangor, Malaysia (n = 126)

[**insert** Figure 2A]

At the time of the workplace cluster, mass gatherings were not banned. A second cluster was subsequently detected after a religious mass gathering in Kuala Lumpur of more than 10 000 people between 28 February and 2 March 2020, resulting in 559 COVID-19 cases in Selangor among attendees, their families and social contacts (**Fig. 2B**). Further links were made to a wedding on 6 March and the transfer of students from a school near the mass gathering location to another school in Selangor on 12 March. The earliest onset of disease after the latter event was on 26 February in a cook at the school in Selangor, who also attended the mass gathering.

**Figure 2B:**
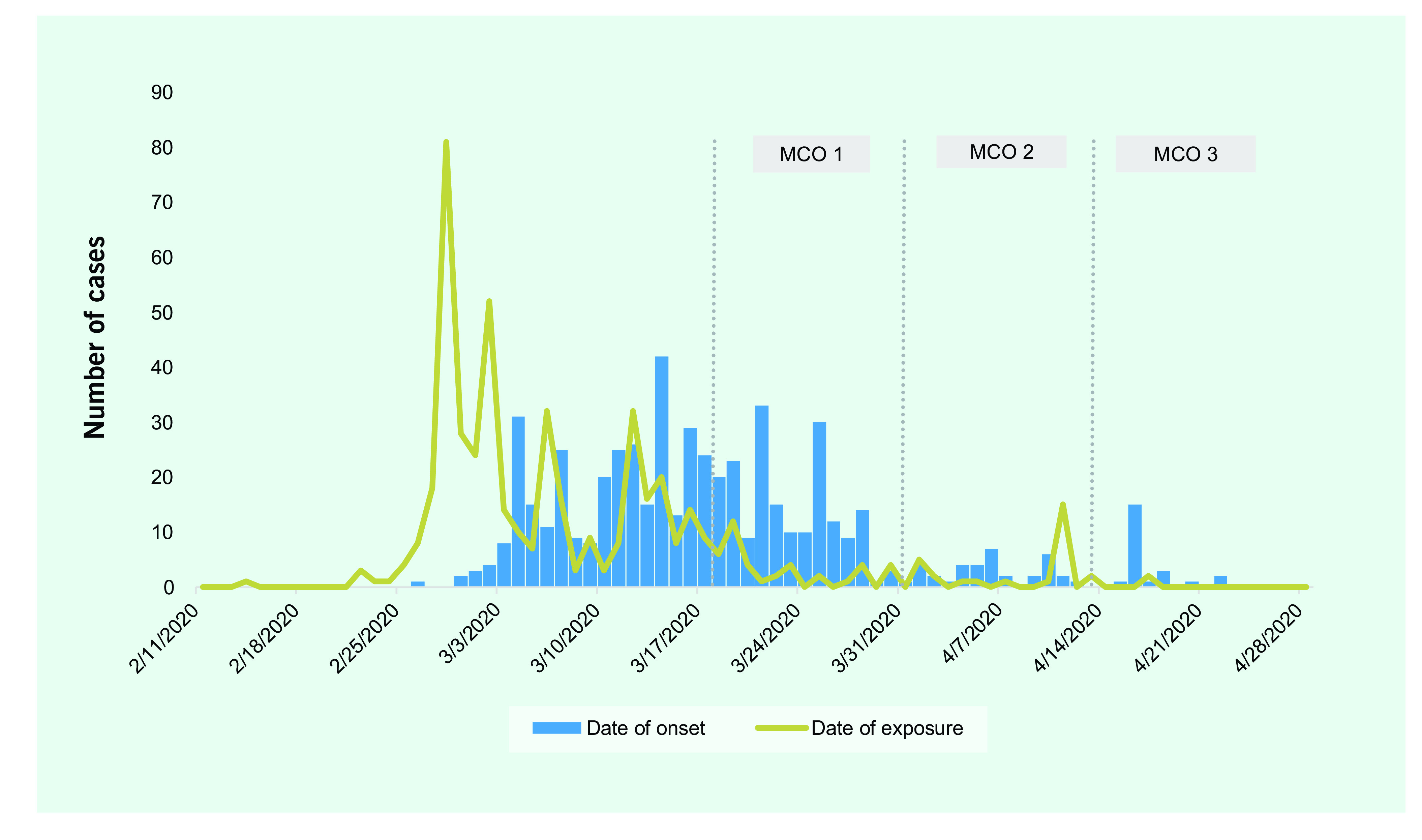
Distribution of cases by date of illness onset and date of exposure in a cluster in Selangor after attendance at a mass religious gathering in Kuala Lumpur (n = 559)

[**insert** Figure 2B]

On 18 March, the first 14-day MCO was initiated, which prohibited public movement, including interstate and international travel and mass gatherings for religious, sports, social and cultural activities throughout the country. Businesses and services deemed non-essential, schools, universities and government offices were closed, and people were urged to work from home. Only essential services such as food and health care could operate, with strict operating procedures that ensured physical distancing and screening for fever. A second MCO was implemented from 1 April to 14 April. In addition, an enhanced MCO was enforced in certain locations with established large clusters, where all movement was restricted. Comprehensive testing of all residents for SARS-CoV-2 was conducted; residents and visitors in the area were forbidden to leave their homes, and all roads into the enhanced MCO area were blocked. Residents were provided with adequate food and medical supplies by authorities, with special arrangements to address any additional needs.

During the first 14 days of the first MCO, the number of COVID-19 cases decreased by 12.8%, with a further decline of 71% after the second and 72% after the third MCO. The number of imported cases fell after implementation of international travel restrictions during the first MCO and had almost disappeared by the third. Most unlinked cases were reported before and throughout the first MCO and had also fallen to almost 0 during the third.

## Discussion

Lack of pharmacological treatment and vaccines against COVID-19 meant that public health and social measures were the mainstay of the initial COVID-19 response. Selangor initially adopted contact-tracing, isolation of cases and quarantine of contacts to manage the outbreak but added MCOs with closure of schools, universities and non-essential businesses and services. The MCOs appear to have flattened the epidemic curve. A modelling study conducted in the United Kingdom that included various transmission routes and mitigation measures suggested that lockdowns alone, particularly if short, will not eliminate transmission and that a combination of stricter measures is required. (*3*)

One of the main public health measures used to reduce importation of cases of COVID-19 was thermal body scanning and health declarations at points of entry. However, asymptomatic and presymptomatic cases can effectively shed the virus (*2*) and are unlikely to be detected by screening at points of entry. One study showed that half of infected travellers are not detected during airport screening. (*4*) In the initial workplace cluster in Selangor, the index case was an imported case that had not been detected at the point of entry. With a substantial proportion of asymptomatic cases (30%), additional control methods are required.

The initial workplace cluster in Selangor was successfully interrupted through the public health measures of contact-tracing, isolation of all confirmed cases and home quarantine of all contacts. Contact-tracing has been a key public health response during previous pandemics of influenza and other communicable disease outbreaks, as it identifies potentially infected individuals before symptoms emerge. (*5*) If conducted promptly, contact-tracing can prevent onward transmission from secondary cases. (*6*) Although contact-tracing can be highly effective for the control of COVID-19, it places substantial demands on the public health authorities, as reported in other studies. (*7*)

The second cluster, arising from the mass gathering in Kuala Lumpur, involved cases all around the country as attendees dispersed to their respective states. In Selangor, contacting and then testing the large number of potential contacts from this event stretched the state’s capacity, and the response to the first cluster of 126 cases could not be replicated for the second cluster of 559 cases. Therefore, the first MCO was enforced, resulting in a reduction in the number of new cases, which continued during the second and third MCOs. Had mass gatherings been prohibited during the earlier phase of COVID-19, this outbreak could have been prevented. However, as a result of this cluster, MCOs were identified as a useful, practicable control measure, which can be implemented intermittently as required.

The objective of the MCO was to reduce contact of potential cases with others, thereby averting widespread community transmission and preventing the health care system from being overwhelmed by an influx of new patients. Extension of the MCO was made possible by government support through an economic stimulus package to ease the burden on businesses and individuals of the economic downturn. (*8*) Although costly, MCOs were seen to slow the epidemic. An interrupted time-series study in Hubei and Guangdong provinces in China before and after lockdown showed a significant reduction in the incidence of cases, indicating the effectiveness of lockdown in containing the outbreak. (*9*) A local modelling study with various contact rates during the phases of MCO found that MCO implementation flattened the epidemic curve, (*10*) and the effectiveness of lockdown in reducing transmission rates has been shown by modelling elsewhere. (*3*) It should be noted, however, that the decrease in the number of COVID-19 cases in Selangor might have also been the effect of the combined prevention strategies, such as isolation, quarantine, travel bans and closure of schools and universities, and not the MCO alone.

The study has several limitations. As Selangor implemented several public health measures concurrently, the relative impact of each intervention could not be evaluated. Nevertheless, our data show a temporal association between trends in the epidemic curve and MCO implementation. Additionally, we did not directly assess changes in human contact behaviour before and during the MCO.

Our study results support the conclusion that MCOs, in conjunction with other public health and social measures, played a key role in controlling the spread of SARS-CoV-2 in Malaysia.
